# Associations between bullous pemphigoid and hematological diseases: Literature review on mechanistic connections and possible treatments

**DOI:** 10.3389/fimmu.2023.1155181

**Published:** 2023-03-08

**Authors:** Yuyan Yang, Wenling Zhao, Nan Yang, Shengnan Cui, Hongzhong Jin, Li Li

**Affiliations:** ^1^ Department of Dermatology, State Key Laboratory of Complex Severe and Rare Diseases, Peking Union Medical College Hospital, Chinese Academy of Medical Science and Peking Union Medical College, National Clinical Research Center for Dermatologic and Immunologic Diseases, Beijing, China; ^2^ Department of Dermatology, Shunyi Maternal and Children’s Hospital of Beijing Children’s Hospital, Beijing, China; ^3^ Department of Pharmacology, Institute of Basic Medical Sciences, Chinese Academy of Medical Sciences & School of Basic Medicine, Peking Union Medical College, Beijing, China

**Keywords:** bullous pemphigoid, hematological diseases, mucosal hematoma, eosinophil, corticosteroid

## Abstract

Bullous pemphigoid is an autoimmune blistering disorder that primarily occurs in elderly patients. Reports indicate that BP coexists with various hematological diseases, including acquired hemophilia A, hypereosinophilic syndrome, aplastic anemia, autoimmune thrombocytopenia, and hematological malignancies. Early identification of these comorbidities contributes to a better control and reduced mortality. This article details the atypical clinical manifestations of BP when associated with hematological diseases, specific diagnostic strategies, underlying mechanistic connections, and possible treatments. Cross-reactivity between autoantibodies and exposed abnormal epitopes, shared cytokines and immune cells, together with genetic susceptibility are the most common connections between BP and hematological diseases. Patients were most often successfully treated with oral steroids combined with medications specifically targeting the hematological disorders. However, the individual comorbidities require specific considerations.

## 1 Introduction

Bullous pemphigoid (BP) is an autoimmune blistering disease which is characterized by the presence of cutaneous bullae and autoantibody deposition at the epithelial basement membrane zone (1). BP affects predominantly elderly people while its mortality rate increases with each increasing decade of life (2). In European and Asian populations, the incidence of BP is 10.3 and 5.2 per million, respectively (3). Because of the development of improved diagnostic assays as well as aging populations, the annual incidence of BP is increasing (4). Classical manifestations of BP include tense blisters on the extremities and trunk with less mucosal involvement than the similar autoimmune blistering disease, pemphigus vulgaris (5, 6).

Autoantibodies against the auto-antigens BP180 (180kDa) and BP230 (230kDa) have been identified in BP patients. Epitope mapping indicated a relationship between autoantibody specificity and disease phenotype (7). The non-collagen extracellular domain of BP180, termed NC16A, is the primary binding epitope by anti-BP180 antibodies, correlated with typical severe erythematous inflammation and blister formation (8, 9). Noninflammatory bullous pemphigoid manifested as reduced erythema and sparse periblister eosinophilic infiltration is thought to be caused by autoantibodies targeted the midportion of BP180, probably associated with dipeptidyl peptidase- IV inhibitors (9). Furthermore, autoantibodies against multiple extracellular domains of BP180 may increase the morbidity of mucosal lesions (10). The typical binding site for BP230 is the globular C-terminal domain (11). BP severity and activity are reported to correlate directly with levels of anti-BP180 autoantibodies, but no consistent correlations with anti-BP230 autoantibody titers have yet been identified (1). However, because the phenomenon of epitope spreading occurs at an early stage of disease when BP230 is exposed, anti-BP230 autoantibodies also contribute to the development of skin lesions (11). IgG4 is the predominant subclass of BP autoantibodies, followed by IgG1 and IgG2. IgA and IgE autoantibodies directed at the basement membrane zone may also be present in BP (12).

BP has been reported in patients with concomitant hematological disorders, especially in older populations. Dysregulation of coagulation system results in abnormal bleeding issues, acquired hemophilia A and autoimmune thrombocytopenia (13, 14). Combination of these coagulation disorders with BP were reported to occur in elder populations, presenting as atypical hemorrhagic skin lesions. Changes in hematocytes and leukocytes usually destabilize the circulation and immune system, thus BP patients diagnosed with hypereosinophilic syndrome and aplastic anemia requires more concerns (15, 16). Hematological malignancies such as myelodysplastic syndrome and chronic lymphocytic leukemia are reported to be significantly associated with BP compared with other neoplasms (17–19). Acquired hemophilia A, autoimmune thrombocytopenia, hypereosinophilic syndrome and aplastic anemia are characterized by obscure onset and robust diagnostic assays. Early identification of these comorbidities contributes to a better control. On the other hand, detection of myelodysplastic syndrome and lymphoproliferative disorders in BP patients may conduce to better prognosis and mortality reduction.

The etiologies of BP and hematological disorders were proposed to be related rather than independent. Cross-immunoreactivity, specific cytokines and genetic predisposition were proposed to play important roles (5, 13). Most patients’ skin conditions were influenced by the additional hematological diseases. Hematological comorbidities and clinical features such as hematoma and eosinophilic dermatitis-like lesions make diagnosis of BP challenging without further examination. Previous studies of patients with BP and comorbidities such as neurologic diseases, neoplasms, cardiovascular diseases found that if these patients did not receive timely treatment, their mortality rates exceeded those of patients diagnosed with BP alone (18, 20). Thus, we assume that cases of BP associated with hematological diseases require more attention.

In this review, we discuss the atypical clinical manifestations associated with comorbidities, to aid diagnosis of BP in patients with hematological diseases. Furthermore, we describe the mechanisms present in BP cases complicated with hematological diseases. Finally, we present possible treatment strategies to improve the prognosis of these cases (concluded in **Table 1**). Details of all cases mentioned in this review were recorded in **Table 2**, especially the complex manifestations and therapeutic effects.

**Table 1:** Characteristics of patients diagnosed with bullous pemphigoids and hematological comorbidities.

Comorbidities	Atypical Manifestations	Possible mechanisms	Treatment
**Acquired hemophilia A**	cutaneous-mucosal bleeding, hematoma, hemarthrosis	cross-immunoreactivity, immunogenic susceptibility	corticosteroid (first line), immunosuppresive drugs, FVIII replacement therapy and bypassing agents
	mild skin lesions	coagulation cascade disruption	
**Hypereosinophilic syndrome**	eosinophilia dermatitis-like, pruritic exzema-like	protease, IL-5 and veotaxin, breakpoint in 20q11 region of BRK gene	imatinib mesylate, eotaxin and IL-5 reducing therapy
**Aplastic anemia**	typical	Th17 cells	oral prednisolone along with cyclosporine
**Autoimmune thrombocytopenia**	typical	HLA-DR3 and DR4	corticosteroid
**Hematological malignancies**	typical, debilitation, mucosal bullae	neoplastic autoantibodies, cross-immunoreactivity, tumor-induced epidermal lesion	high dose oral prednisolone and azathioprine, chlorambucil, rituximab

IL-5, interleukin-5; FVIII, factor VIII; Th17 cells, T helper 17 cells; HLA, human leukocyte antigen.

**Table 2:** Reported cases of bullous pemphigoid associated with various hematological diseases.

Comorbidity	Author	Year	Gender/age †, years	BP Onset	Clinical Features	Response to treatment of BP	Treatment of Co-disease	Response to treatment of Co-disease
**AHA**	Ma et al. (20)	2021	M/63	7 months before AHA	Blisters	Relapsed after CS tapering; No response to CTX; Resolved with low-dose CS, RTX and rFVIIa	CS, RTX, rFVIIa	Complete remission
					Ecchymosis and swelling of lower limbs			
					Large hematoma on right shoulder			
					Intracranial hematoma and right-sided pleural effusion			
	Braganca et al. (21)	2021	M/74	8 months before AHA	No blisters	Relapsed after CS tapering;Resolved with CS and FEIBA	CS, FEIBA	Complete remission
					Multiple ecchymosis on arms, thorax and jaw			
					Tense muscular hematoma on right thigh			
	Fakprapai et al. (12)	2019	F/68	11months before AHA	Bullae on trunk and extremities	Resolved with CS, nicotinamide	CS, CTX, FEIBA	Complete remission
					Large hematoma on right buccal mucosa			
	Binet et al. (23)	2017	M/75	21 months before AHA	Blisters especially on flexural areas	Controlled with CS, AZA/MMF	CS, RTX, rFVIIa	Complete remission
					Swelling of right knee and two wrists			
					Recurrent subconjunctival hemorrhages and epistaxis			
	Aljasser et al. (24)	2014	M/73	1 months before AHA	Blisters	Minimal response with CS; Controlled with RTX and CTX	CS, IVIg, CTX, RTX, rFVIIa, FEIBA	Complete remission
					Upper gastrointestinal bleeding and hemoptysis			
					Retroperitoneal hematoma.			
	Makita et al. (25)	2013	F/80	12 months before AHA	No blisters	Resolved with CS	CS	Complete remission
					Subcutaneous bleeding of arms			
					Gingival hemorrhage			
	Ammannagari et al. (26)	2013	M/69	1 months before AHA	Erythematous blisters and ecchymoses on arms	Resolved with CS	CS, RTX, rFVIIa	Complete remission
	Qiu et al. (27)	2012	F/60	Concurrently with AHA	Hemorrhagic bullae on extremities and trunk	N/D	CS, CTX, IVIg, rFVIIIa	Complete remission
					Hematoma in base of tongue			
	Zhang et al. (28)	2012	F/88	4 months before AHA	Blistering eruption	Not improved with CS; Controlled with CS and RTX	mPSL, RTX	Complete remission but died with severe pneumonia and multi-organ failure
					Large hematoma on back			
					Extensive ecchymosis			
	Nguyen et al. (29)	2012	F/49	4 months before AHA	Bullae on trunk and extremities	Minimal response to CS and IVIg	CS, CTX, FEIBA	Complete remission
					Intense pruritus and worsening bullous rash on lower legs			
	Kluger et al. (30)	2011	M/72	9 months before AHA	Itchy, blistering skin eruption	Resolved with MTX and topical CS	CS, RTX, rFVIIa	Complete remission
	Chen et al. (31)	2010	M/24	2 years before AHA	No blisters	Resolved with CS	mPSL, CTX, CS, RTX, rFVIIa, Plasmapheresis	Improved after 2 months
					Bruising, swelling and purpura over back and abdomen			
	Maczek et al. (32)	2002	M/47	Concurrently with AHA	Extensive hemorrhagic erosions of oral, genital and nasal mucosa Subcutaneous hematomas on abdomen	Resolved with MTX and CS	Plasmapheresis, CS	Stable remission
**ATP**	Taylor et al. (13)	1993	M/63	18 months before ATP	Blisters on upper body	Resolved with CS	N/D	N/D
	Aoki et al. (33)	1990	M/25	Concurrently with ATP	Blisters on trunk and legs, some are hemorrhagic	Minimal response to CS and SUL	N/D	N/D
					Oral ulcer and nasal erosion			
**HES**	Wang et al. (34)	2017	F/73	11 months before HES	Blisters and erythema on trunk	N/D	CS, MTX and IFN-α	Complete remission
					Funicular and whorled hyperpigmentation on trunk			
					Papules or nodules on extremities			
	Hofmann et al. (14)	2007	M/70	7 months before HES	Blisters on feet	Slight improvement with topical CS, AZA and FEX	IM	Complete remission
					Pruritic eczema-like skin lesions			
					Erythematous papules on trunk			
					Excoriations on legs			
	Belgnaoui et al. (35)	2002	M/58	N/D	Generalized bullae	Improved with CS	CS	Resolved
**AA**	Fujimura et al. (15)	2012	M/58	After 20 years AA	Generalized large bullae and erosion on erythema	Controlled with CS and CsA	CS and CsA	Complete remission
**MDS**	Lee et al. (16)	2011	M/67	After 3 months MDS	Hemorrhagic bullae on extremities and oral mucosa	Controlled with CS and AZA	Blood transfusions	Resolved
	Bauduer et al. (36)	1999	F/82	After 6 months MDS	Generalized blisters on erythematous plaques	Not improved with CS and OX	HU	Died after 10 days
	Modiano et al. (37)	1997	M/86	After 11 years MDS	Generalized hemorrhagic blisters on erythema	Resolved with CS and HU	N/D	Died due to hematological disease
**CLL**	Ivars et al. (38)	2015	M/79	After several months CLL	Generalized tense blisters and erosions	Resolved with CS, dapsone and RTX	RTX	Complete remission
					Severe vesicular and erosive lesions on oral mucosa			
	Kassim et al. (39)	2015	F/72	After 2 years CLL, coexisted with UV	Blisters on thighs	Not improved with lymecycline; Controlled with CLB but relapsed; Resolved with RTX	CLB, R-FC therapy (RTX, fludarabine and CTX)	Complete remission
					Generalized discrete urticated papules and plaques			
	Kakurai et al. (40)	2009	M/72	After 1 year CLL, coexisted with PNP	Violaceous erythema and tense bullae on trunk and extremities	Resolved with CS and CTX	COP therapy (CS, vincristine and CTX)	Died due to aspiration pneumonia after 40 days
					Sever erosions on lip, tongue and palate			
					Hyperemic conjunctivae			
	Saouli et al. (41)	2008	F/58	N/D	Vesiculobullous lesions on trunk and all extremities	Not improved with CS; Resolved with RTX	RTX	Complete remission
		2008	F/78	N/D	Vesiculobullous lesions on trunk and all extremities	Not improved with CS; Resolved with RTX	RTX	Complete remission
	Ameen et al. (42)	2000	F/77	After several months CLL	Generalized vesicles and small blisters	Not improved with dapsone and antibiotics; Improved with CLB; Resolved with CTX	CS and CLB	Resolved
					Large erythematous annular plaques			
					Arcuate lesions with crusting			
	Misery et al. (43)	1999	M/81	Concurrently with CLL	Typical BP lesions	N/D	N/D	N/D
	Su et al. (44)	1994	M/67	After 6 months CLL, coexisted with PNP	Flaccid and tense bullae in adjacent sites and distant areas	Controlled with CS; Improved with AZA	CS, CTX, plasmapheresis	N/D
					Eroded flaccid bullae on glans penis			
					Confluent bullae and erosions on buccal mucosae and lips			
	Goodnough et al. (45)	1980	F/81	Concurrently with CLL	Generalized blisters and tense bullae in various stages	Not improved with CS; Resolved with CLB	CLB	N/D
	Cuni et al. (46)	1974	F/72	After 5 months CLL	Generalized tense bullae with erythematous bases	Not improved with CS; Resolved with CS and CLB	CLB	Died after 1 month
					Hemorrhagic bullae			

*The cases are presented in order of publication date.

†Gender: M(ale)/F(emale).

BP, bullous pemphigoid; AHA, acquired hemophilia A; ATP, autoimmune thrombocytopenia; HES, hypereosinophilic syndrome; AA, aplastic anemia; MDS, myelodysplastic syndrome; CLL, chronic lymphocytic leukemia; AZA, azathioprine; CTX, cyclophosphamide; CS, corticosteroid; RTX, rituximab; CsA, cyclosporin; FEIBA, factor VIII inhibitor bypassing agents; IVIg, intravenous immunoglobulin; rFVIIa, recombinant human factor VII; rFVIIIa, recombinant human factor VIII; MMF, mycophenolate mofetil; mPSL, pulse methylprednisolone; MTX, methotrexate; SUL, sulfamethoxypyridazine; HU, hydroxycarbamide; OX, oxacilline; FEX, fexofenadine; IM, imatinib mesylate; IFN-α, interferon alfa-2b; UV, urticarial vasculitis; PNP, paraneoplastic pemphigus; CLB, chlorambucil; N/D, not described.

## 2 Acquired hemophilia A

Acquired hemophilia A (AHA) is a rare autoimmune disease with a high mortality rate ranges from 17-22% that results from the development of circulating autoantibodies against the endogenous coagulation factor VIII (47). AHA is characterized by spontaneous hemorrhage and excessive bleeding in the absence of a family history or childhood experience of disordered bleeding. Although activated partial thromboplastin time (APTT) is prolonged, thrombin times, prothrombin times, platelet count and function are usually normal (48).

Approximately 50% of AHA patients are concomitantly diagnosed with other diseases, especially autoimmune diseases including systemic lupus erythematosus, rheumatoid arthritis and BP (23). The combination of BP and AHA is relatively rare, with no more than 30 documented cases reported globally, and most of these patients had abnormally bleeding skin lesions (23). AHA often occurs in BP patients aged 24 to 88 years and is present in 68% patients aged over 65 years at diagnosis, with no sex predisposition (23). BP is typically diagnosed prior to AHA onset or they occur simultaneously, with the mean time between the diagnosis of the two diseases being 6 months (23). In none of the current reports does AHA occur prior to BP diagnosis.

Severe cutaneous-mucosal bleeding is seldom reported in BP patients (6). However, these symptoms usually indicate that there might be complicating AHA. Prior to the onset of AHA, hemorrhagic blisters rarely appear and APTT remains normal (49). Only after the development of AHA will extensive hemorrhagic bullae and large hematomas involving mucous membranes begin to appear (13). Hemarthrosis, which is not characteristically seen in AHA patients, has also been reported in a comorbidity case with BP (23). However, a minority of patients were reported to have unexpected mild skin lesions despite their high BP180 autoantibody titers (23). Because of this intersection of manifestation and immunological index, diagnosis of these patients could easily be missed and requires more clinical attention.

Apart from the clinical manifestations, diagnosis of the combination of AHA and BP further depends on laboratory tests. High anti-BP180 titers, prolonged APTT and increased FVIII inhibitor levels occurred simultaneously in an elderly patient as evidence of the comorbidity diagnosis (23). For most patients, laboratory tests indicate a parallel course of these two diseases. During remission of the two concurrent diseases, the eradication of FVIII inhibitors increased FVIII levels and shortened APTT, while anti-BP180 and BP230 IgG titers also decreased simultaneously (23).

Intrinsic risk factors for both AHA and BP might exist as there are abnormal clinical manifestations and parallel remission. One potential mechanism for the onset of comorbid disease is cross-reactivity between autoantibodies against the BP180 NC16A domain and factor VIII A2. This theory is strongly evidenced by sequence homology between the two binding domains (23). Autoantibodies found in patients with either disease predominantly belong to the IgG4 subtype, while a minority are of the IgG1 subtype (50). Though the case reported by Makita denied cross-activity between factor VIII and BP180, this negative result might be interfered by immunosuppressive therapies (25). However, no regions of high similarity have been identified between the NC16A domain of BP180 and factor VIII A2 (49). Furthermore, atypical clinical manifestations were also suggested to be associated with autoantibody cross-reactivity. Serum IgG extracted from a 47-year-old patient with AHA and rather mild BP targeted the mid-portion of extracellular domain of the BP180 antigen, which was reported to be related with reduced erythematous skin lesions and increased mucosal involvements (32).

A 78-year-old patient with BP and AHA was reported in 2006 and his medical history also included rheumatoid arthritis and vitiligo (49). His comorbidity was noted following persistent bleeding from buccal hemorrhagic swelling. As for those patients diagnosed with several simultaneous autoimmune diseases, studies inferred that there might be an underlying immunogenetic susceptibility to autoimmune diseases in them (49). BP patients diagnosed with other immunological abnormalities might have severe cutaneous-mucosal symptoms. However, no such genes have been discovered to date.

The reasons why mild cutaneous symptoms coexist with high autoantibody titers in a few patients with comorbidities remain unclear. According to previous researches, we inferred that this abnormality might be partially explained by a disruption in the coagulation cascade in patients with this comorbidity. Excessive coagulation activation has been demonstrated in BP and is associated with higher thrombotic risk (51). Infiltrating T cells, predominantly T helper type 2 (Th2) cells, in BP skin lesions can produce interleukin (IL)-5 and eotaxin to recruit and activate eosinophils (52). Eosinophils store and rapidly transfer tissue factors (TF) to the cell membrane (52). This signaling process leads to increased F1+2 and D-dimer levels, which initiate the coagulation cascade (51). This hypercoagulability usually contributes to tissue damage and blister formation in BP patients, thus forming a vicious cycle between cutaneous lesions and thrombosis.

Poor prognostic factors include elder age, other comorbidities, high anti-BP180 and anti-BP230 titers as well as high factor VIII inhibitor titers (53). It was reported that a patient with BP-induced AHA developed intracranial hematoma and hemothorax during the acute phase (21). Infections in continuously bleeding mucosal lesions should also be prevented. Thus, treatment of coexisting BP and AHA is primarily focused on autoantibody eradication and bleeding remission (13, 27). Immunosuppressive drugs have shown some benefit for both AHA and BP. The first-line therapy for this comorbidity is oral corticosteroids alone or with cyclophosphamide (13). Tapering of corticosteroids should be elaborated to avoid severe relapse (22). Rituximab was used in cases reported in 2012 and 2021 with successful disease management and is often used as a second-line therapy (13, 20, 21, 28). However, as another newly developed biologic, omalizumab was suspected a causality in AHA onset (54). Other treatments include cyclophosphamide, high-dose immunoglobulins and immunoabsorption (13, 23, 55). Tapering of immunosuppressive agents is more prudent because immune reconstitution inflammatory syndrome presenting as AHA has been reported in BP patients (56). Immune recovery, which occurs rapidly after inadequate withdrawal of the drug, may trigger subsequent AHA recurrence. Because of the persistent presence of circulating factor VIII autoantibodies, factor VIII remains insufficient. Thus, triggering the extrinsic coagulation pathway might be a targeted substitution for the disabled intrinsic pathway. Factor VIIa is a key component of the prothrombin activation complex in the bypass coagulation pathway. Consequently, recombinant activated factor VII and activated prothrombin complex concentrate were often prescribed. However, this dose should not exceed 200 units/kg daily as there is an increased risk of venous thromboembolism (13). When there was no elevated FVIII inhibitor titer, human FVIII replacement therapy may also be effective (13). Bypass therapies, including recombinant activated factor VIIa, activated prothrombin complex concentrate, and neutralization therapy mainly composed of factor VIII concentrates aim to control bleeding and maintaining effective hemostasis (25).

Non-specific adverse effects (e.g., infection, sepsis, neutropenia) are likely to occur in BP patients with comorbid AHA, especially those with severe skin lesions. Cutaneous relapses were also often seen after drug discontinuation (57, 58). Thus, an appropriate tapering dose should be administered for the treatment of this comorbidity.

Collectively, when BP patients developed extensive hemorrhagic bullae, large mucosal hematoma or even hemarthrosis, a combination with AHA should be investigated. BP180 titers only correlates partially with disease severity, with the linear correlation coefficient reported as 0.6 (59). However, if a patient with mild skin lesions and abnormally high autoantibody titers concomitantly complained about predisposed to easy bleeding, potential presence of comorbid AHA should be noticed. Clinicians are strongly advised to examine APTT and factor VIII inhibitors to diagnose the comorbidity and further adjust the current treatments.

## 3 Autoimmune thrombocytopenia

Autoimmune thrombocytopenia (ATP) is an autoimmune disease characterized by an autoantibody-mediated decrease in platelets (60).

Only a few patients have been reported to develop ATP after BP (14, 33). The interval course between BP and ATP ranged from 18 months to 3 years. Additionally, cutaneous manifestations were typical, presenting as pruritic bullous erythema on trunk and extremities.

Therapeutic agents, such as prednisolone and sulfamethoxypyridazine, have been associated with thrombocytopenia. Drug molecules are supposed to induce thrombocytopenia *via* direct cytotoxic effects on megakaryocytes and platelets, which leads to dysfunctional thrombopoiesis and increased platelet destruction (61). However, discontinuation of BP treatment did not resolve the thrombocytopenia (14). Thus, researchers have inferred that medications curing BP might be just triggers rather than the direct cause of the development of antiplatelet autoantibodies. Genetic predisposition may additionally play an important role as HLA-DR3 and DR4 haplotypes were found in a patient with both BP and autoimmune thrombocytopenia (14). Systematic corticosteroid therapy was suggested for skin lesions treatment because of its satisfactory therapeutic effect (14).

To summarize, it is important to monitor the development of thrombocytopenia after BP remission.

## 4 Hypereosinophilic syndrome

Hypereosinophilic syndrome (HES) is a myeloproliferative disorder characterized by idiopathic eosinophilia of at least 1500 cells/μl for more than 6 months with cutaneous or systemic involvement. Intense eosinophilic infiltration at the dermal–epidermal junction can be seen in skin biopsies (62). As a clonal hematopoietic disorder, two chromosomal abnormalities involving tyrosine kinases have been identified. Most HES patients carry an interstitial deletion on chromosome 4q12, resulting in the formation of a fusion gene termed *FIPL1-PDGFRA*. This gain-of-function mutation leads to the expression of constitutively active tyrosine kinase (63, 64). Deletion of the 20q11 region disrupts the nonreceptor tyrosine kinase gene *BRK*, which also causes an abnormal activation of tyrosine kinases (15). Both mutations lead to excessive peripheral and tissue eosinophils.

Comorbidities between BP and HES are extremely rare, with only six cases reported (15, 34, 35, 63). Because HES is usually diagnosed during routine follow-up rather than at the time of onset, determining the onset order of these two diseases is challenging. However, levels of eosinophils appeared to increase in more than 50% of BP patients, which could mask the coexisting HES if they exceeded 1500 cells/μl (65). Consequently, this comorbidity is particularly difficult to diagnose.

The clinical manifestations of patients with comorbid BP and HES were reportedly atypical. Skin lesions in patients with both BP and HES presented as eosinophilia dermatitis-like or pruritic eczema-like patterns instead of bullae (15, 34). These atypical manifestations often make the diagnosis of BP challenging. Direct immunofluorescence results have indicated that IgG or C3 deposition at the basement membrane zone and detection of BP180 antibodies could help avoid misdiagnosis. Furthermore, in patients with typical BP cutaneous symptoms, anomalously high levels of eosinophils may also be identified.

**Figure 1:**
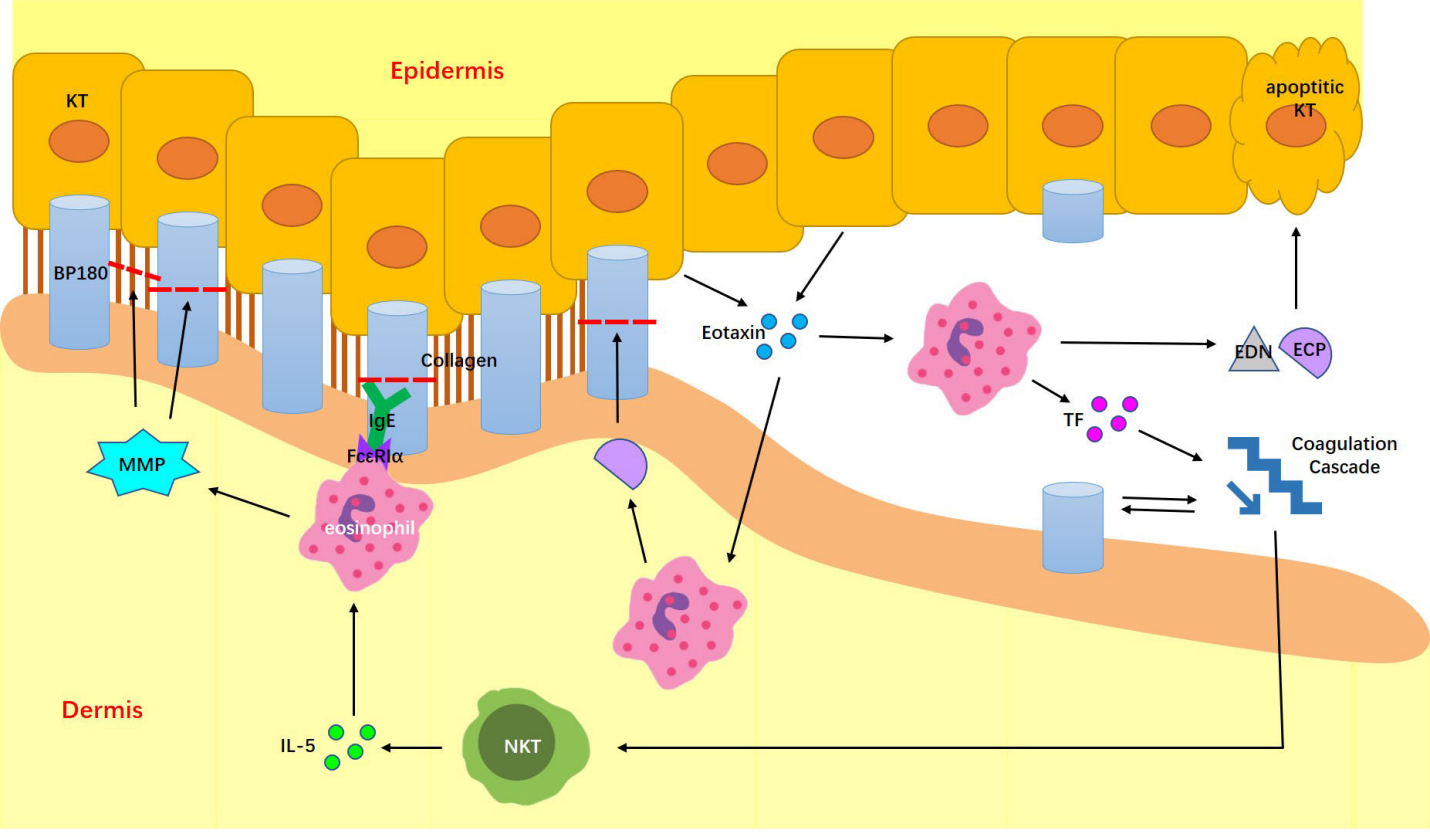
Possible mechanisms of BP combined with HES. IL-5, interleukin-5; MMP9, matrix metalloproteinase 9; EXM, extracellular matrix; ECP, Eosinophil cationic protein; EDN, eosinophil-derived neurotoxin; TF, transfer tissue factor.

Eosinophils play an important role in BP pathogenesis (**Figure 1**). They predominantly infiltrate the dermal region and degranulate, releasing proteases, which induce the skin lesions. One of the most important proteases is matrix metalloproteinase 9 (MMP9), also known as gelatinase, which is capable of degrading extracellular matrix proteins and BP180 (1, 66). Hence, collagen fibers are degenerated and hemidesmosomes are disrupted. Eosinophil cationic protein (ECP) and eosinophil-derived neurotoxin (EDN) are ribonucleases produced by eosinophils, which trigger the apoptosis of keratinocytes around BP lesions. ECP also induces basal keratinocyte detachment (67). Through the activity of the ribonucleases, eosinophils contribute to dermal–epidermal separation and ultimately, blister formation (1). BP patients with comorbid HES have higher levels of blood eosinophilia, and thus may present with more severe skin lesions. However, although the six reported cases had more severe and extensive pruritus, the shape of bullae appeared to be intact without erosions or exudation, potentially because of limited eosinophil infiltration in the dermis and epidermis.

BP triggers eosinophil accumulation mainly through the actions of cytokines, chemokines and autoantibodies. Natural killer T cells located in lesions and eosinophils gathering around BP blisters express excessive IL-5, which further contributes to eosinophil development, release, and degranulation. This mechanism is evidenced by a correlation between IL-5 expression, eosinophil activity and BP blister formation (68). Conversely, eotaxins, the major chemotaxin for eosinophils, were reportedly expressed in the epidermal keratinocytes around BP blisters. This abnormally increased release of eotaxins was possibly triggered by ECP and EDN (67). Eotaxins could recruit further eosinophil infiltration to the blisters. Furthermore, because eosinophils in BP patients were shown to express the high-affinity IgE receptor FcϵRIα, they may bind and be triggered by BP autoantibodies of the IgE subtype (67). The erythematous urticarial phenotype of BP is associated with increased IgE antibody levels (69).

Although elevated eosinophils play an important role in both BP and HES, these cases were shown to be comorbidities rather than complications (15). First, typical BP patients usually have lower levels of eosinophils than HES patients. Furthermore, no specific first-line medications used to treat BP are known to induce reactive eosinophilia. Moreover, the cause of HES is clonal proliferation of eosinophils.

This comorbidity was reportedly treated by imatinib mesylate, a novel tyrosine kinase inhibitor (15, 63). Imatinib mesylate is not a common drug for BP treatment; however, it still led to durable remission of both skin lesions and hypereosinophilia (15). Therapies aim to reduce eotaxin and IL-5 production to within normal ranges may also resolve blister formation and normalize eosinophil counts in BP patients diagnosed with HES. Simultaneous remission of these two diseases further supports the potential relationship between BP and HES.

In summary, in a BP patient presenting with eosinophilia dermatitis-like or pruritic eczema-like skin lesions who also has abnormally high eosinophilia, HES should be considered. In case that symptomatic therapy is unsatisfactory, we strongly recommend testing anti-BP180 titers to help rule out BP and HES comorbidity in HES patients with recalcitrant blood eosinophilia or refractory lesions.

## 5 Aplastic anemia

Aplastic anemia is an immune-mediated disease characterized by peripheral blood pancytopenia and bone marrow hypoplasia (70). Hematopoietic cells are actively destroyed by effector T cells, which results in a prominent decrease in the numbers of white blood cells, red blood cells and platelets. High levels of platelet-associated IgG have also been recognized (16).

Only one case has been reported with both BP and aplastic anemia (16). The aged patient had a 20-year history of uncured aplastic anemia and developed pruritic bullous erythema over his trunk and extremities. Although his clinical manifestations were atypical, the diagnosis was achieved based on high levels of serum BP180 autoantibodies and platelet-associated IgG (16).

IL-17-positive cells have been found infiltrating the BP blisters, which suggests an underlying association with T helper 17 (Th17) cells (16). Th17 cells are critical to the pathogenesis of autoimmune diseases (71). In both BP and aplastic anemia patients, Th17 cells are increased along with a reduction in regulatory T (Treg) cells (72, 73). Th17 cells act destructively in BP by mediating inflammation, while in aplastic anemia, they contribute to the recruitment of Th1 cells and regulation of proinflammatory cytokines in the bone marrow during the early disease stages (72, 73).

The patient with both BP and aplastic anemia was successfully treated with oral prednisolone and cyclosporine. The skin lesions disappeared and the numbers of blood cells returned to normal levels (16).

In summary, when patients with aplastic anemia develop pruritic erythema or bullous lesions, BP should be suspected. Even if anti-BP180 titers turn out to be negative, autoantibodies recognizing other BP related epitopes were recommended to be tested to avoid misdiagnosis.

## 6 Hematological malignancy

Myelodysplastic syndrome (MDS) is a hematological malignancy characterized by a heterogeneous group of malignant hematopoietic stem cell disorders (74).

Only three cases with both BP and MDS were reported (17, 36, 37). Clinical manifestations of patients combined with MDS were more severe than typical BP patients (17). Atypical skin lesions and examination results include multiple bullae on the oral mucosa and autoantibodies against desmoplakin (17, 37). The unusual autoantibodies may arise through recognition of common epitopes between desmoplakin and BP230 as their structures are similar. However, whether they are caused by MDS remains unclear (75).

While both diseases are common among older adults, the relationship between BP and MDS remains unknown. It has been suggested that a cross reaction between tumor-specific antigens and the basement membrane zone might lead to bullae development (76). Modiano reported a case in which tumor CD13+ and CD15+ cells infiltrated the dermal region and led to skin detachment (37). Subsequently, abnormal BP antigens were exposed, which induced further abnormal anti-BP180 expression. Linear IgG and C3 deposition along the basement membrane zone were found surrounding the area (37). Evidence also exists for the cross-reaction theory as BP erupted during the transformation of the previously refractory anemia to subacute myelomonocytic leukemia (36). Furthermore, genetic predisposition and external inducers such as radiation and chemicals are also implicated (77).

Lymphoproliferative disorders are a group of hematological tumors caused by proliferation of clonal malignant lymphoid stem cells (78). Among them, B-cell chronic lymphatic leukemia (CLL) is a the most common form with an annual incidence rate of 5.1/100,000 (79). According to previous studies, the incidence of autoimmune diseases in lymphoproliferative disorders is approximately 8% (42).

A nationwide record-linked study conducted in England (1999-2011) demonstrated elevated risk of BP in patients with lymphoid leukemia compared with a reference cohort (80). Lymphoproliferative disorder-related BP has been reported in 10 cases. All patients developed CLL before or concurrently with BP, with an interval course up to 2 years. Coexistence of paraneoplastic pemphigus was diagnosed in two men (40, 44). Two women also suffered from inflammatory arthritis (39, 45).

Cutaneous lesions in most patients diagnosed with BP and CLL were generalized pruritic blistering skin lesions, similar to those found in classic BP patients. Only a 79-year-old man developed atypical severe oral erosion (38). Discrete polymorphic skin lesions were reported in a 77-year-old man, mainly manifested as large erythematous annular plaques and arcuate lesions (42).

Both characterized by pruritus vesiculobullous lesions and dermal-epidermal detachment, BP-like pattern of Eosinophilic dermatosis of hematologic malignancies (EDHM) closely resembles malignancy-related BP (81, 82). Thus, differential diagnosis is important for precise treatment selection. In EDHM patients, both immunofluorescence and serum tests turned out to be autoantibody-negative against BP180 (83). Immunological examinations can help clinicians distinguish malignancy-related BP from EDHM before medication.

Paraneoplastic pemphigus (PNP) can also present as BP-like pattern while develop autoantibodies targeting BP180 or BP230 in patients with hematologic malignancies. Diagnosis of BP should not be made unless PNP has been excluded based on its diagnostic criteria (84). Recombinant protein containing envoplakin and periplakin are reported to be a sensitive and specific antigen for PNP diagnosis (85).

Lymphoproliferative disorders induce important alternations of the immune system by autoantibodies produced by neoplastic cells (79). It has been confirmed that neoplastic B cells are able to recognize self-antigens (86). Augmentation and hyperactivation of Th2 as well as IL-17-producing cells in B-CLL patients can further promote autoantibody production (79). P105 antigen, the targeted site of non-classic dermal-binding BP, exhibits 70% homology to tumor-associated antigen (39). However, whether the IgG autoantibodies against NC16A domain of BP180 or BP230 were produced by neoplastic cells has not been practically confirmed (40, 44). According to a research conducted by Misery, extracellular immortalized leukemic cells extracted from a patient diagnosed with BP and CLL failed to synthesize anti-230 autoantibody (43). On the other hand, pathogenic anti-BP180 antibodies might also be produced by donor-derived B lymphocytes in patients underwent hematopoietic stem cell transplantation, which need careful surveillance during treatment (87).

Other potential pathomechanisms include auto-reactive T-cell clones, which can be simulated by abnormal antigens presented by neoplastic cells from B-CLL patients (88). Besides, by producing IL-4 and IL-17, dysfunctional T-regulatory cells activated in CLL patients were reported to promote both tumor tolerance and peripheral inflammation (89). Chemotherapeutic agents, such as fludarabine, are also suggested to be associated with abnormal regulation of T-cells (88).

However, there is no direct validation of immunopathological relationship between BP and hematological malignancies, which still need further investigations.

Paraneoplastic dermatoses refer to cutaneous diseases secondary to malignancies (90). Patients with malignancy-associated BP usually have a former oncological medical history prior to BP onset. Furthermore, they usually develop BP at an early age (90). Atypical BP lesion, such as figurate erythema, might be a marker for hematological malignancy (76).

BP in MDS patients was reportedly difficult to control, probably related with immunological disorders synergized with other supposed pathomechanisms, such as hematological treatments, genetic and epigenetic factors. The skin lesions only responded to a combination of high dose oral prednisolone and azathioprine in one patient (17). The remaining two patients died before blister remission was achieved (36, 37).

Firstline treatment for malignancy-associated dermatoses usually referred to systemic steroids and immunosuppressants (79). However, steroids often failed to control BP skin lesions in LL patients (42, 45, 91). Early commencement of chemotherapy for CLL plays an important role in sustained resolution of cutaneous manifestations (39). Chlorambucil is also reported to improve skin lesions while help taper corticosteroids dosage rapidly (42, 45, 46). It is noteworthy that rituximab is specifically effective in both BP and CLL patients, thus result in a better prognosis for CLL-related BP patients (38, 41).

In summary, when BP presents with cachexia or inexplicable refractory course of disease, they should be screened for hematological malignancies. If the underlying tumor is left untreated, BP therapies alone cannot improve prognosis. In rare cases, dermatoses might reflect a potential unfavorable prognosis of the associated hematological malignancy (79).

## 7 Conclusion

The coexistence of bullous pemphigoid and hematological disorders is relatively rare compared with other comorbidities. According to the underlying pathological relationships occurring in both BP and hematological diseases, they may contribute to the symptoms seen in these patients. However, further research is required to illuminate the underlying mechanisms. Atypical clinical manifestations are often seen in patients diagnosed with two combined diseases. Skin lesions that are seldom present in typical BP patients, such as hematomas instead of characteristic bullae, make these comorbidities difficult to diagnose. Overlapping symptoms might result in mutual symptom concealment, where misdiagnosis is probable. Moreover, according to most reported cases, patients with comorbidities were curable following a timely diagnosis and treatment regime. Consequently, these rare comorbidities still require attention. More evidence will enable the prevention, diagnosis, specific treatment and health care of BP combined with hematological diseases.

## Author contributions

LL, HJ, and YY contributed to conception of this review. SC and NY collected relevant resources. YY, WZ and NY performed the statistical analysis. YY and WZ wrote the first draft of the manuscript. YY and LL drew the figure. YY and SC performed the tables. All authors contributed to the article and approved the submitted version.

## References

[1] Miyamoto D Santi CG Aoki V Maruta CW. Bullous pemphigoid. Bras Dermatol (2019) 94(2):133–46. doi: 10.1590/abd1806-4841.20199007 PMC6486083 31090818

[2] Ahmed AR Anwar S Reche PA. Molecular basis for global incidence of pemphigoid diseases and differences in phenutypes. Front Immunol (2022) 13:807173. doi: 10.3389/fimmu.2022.807173 35126393 PMC8813746

[3] Persson MSM Begum N Grainge MJ Harman KE Grindlay D Gran S. The global incidence of bullous pemphigoid: a systematic review and meta-analysis. Br J Dermatol (2021) 21(10):4818–35. doi: 10.1111/bjd.20743 34480482

[4] Egami S Yamagami J Amagai M. Autoimmune bullous skin diseases, pemphigus and pemphigoid. J Allergy Clin Immunol (2020) 145(4):1031–47. doi: 10.1016/j.jaci.2020.02.013 32272980

[5] Moro F Fania L Sinagra JLM Salemme A Zenzo GD. Bullous pemphigoid: Trigger and predisposing factors. Biomolecules. (2020) 10(10):1432–59. doi: 10.3390/biom10101432 PMC7600534 33050407

[6] Chen X Zhao W Jin H Li L. Risk factors for mucosal involvement in bullous pemphigoid and the possible mechanism: A review. Front Med (2021) 8:680871. doi: 10.3389/fmed.2021.680871 PMC8172594 34095183

[7] Hammers CM Stanley JR. Recent advances in understanding pemphigus and bullous pemphigoid. J Invest Dermatol (2020) 140(4):733–41. doi: 10.1016/j.jid.2019.11.005 32200875

[8] Nishie W. Collagen XVII processing and blistering skin diseases. Acta Derm Venereol (2020) 100(5):adv00054. doi: 10.2340/00015555-3399 32039455 PMC9128997

[9] Izumi K Nishie W Mai Y Wada M Natsuga K Ujiie H. Autoantibody profile differentiates between inflammatory and noninflammatory bullous pemphigoid. J Invest Dermatol (2016) 136(11):2201–10. doi: 10.1016/j.jid.2016.06.622 27424319

[10] Di Zenzo G Grosso F Terracina M Mariotti F De Pita O Owaribe K. Characterization of the anti-BP180 autoantibody reactivity profile and epitope mapping in bullous pemphigoid patients. J Invest Dermatol (2004) 122(1):103–10. doi: 10.1046/j.0022-202X.2003.22126.x 14962097

[11] Shih YC Wang B Yuan H Zheng J Pan M. Role of BP230 autoantibodies in bullous pemphigoid. J Dermatol (2020) 47(4):317–26. doi: 10.1111/1346-8138.15251 32048350

[12] Zhou T Peng B Geng S. Emerging biomarkers and therapeutic strategies for refractory bullous pemphigoid. Front Immunol (2021) 12:718073. doi: 10.3389/fimmu.2021.718073 34504496 PMC8421646

[13] Fakprapai W Wattanakrai P. Bullous pemphigoid associated with acquired hemophilia a: A case report and review of the literature. Case Rep Dermatol (2019) 11(2):130–9. doi: 10.1159/000499525 PMC6547275 31182947

[14] Taylor G Venning V Wojnarowska F. Bullous pemphigoid and associated autoimmune thrombocytopenia: two case reports. J Am Acad Dermatol (1993) 29(5 Pt 2):900–2. doi: 10.1016/0190-9622(93)70266-v 8408837

[15] Hofmann SC Technau K Muller AM Lubbert M Bruckner-Tuderman L. Bullous pemphigoid associated with hypereosinophilic syndrome: simultaneous response to imatinib. J Am Acad Dermatol (2007) 56(5 Suppl):S68–72. doi: 10.1016/j.jaad.2006.02.059 17097375

[16] Fujimura T Kakizaki A Kambayashi Y Furudate S Aiba S. Bullous pemphigoid accompanied by aplastic anemia: The induction of IL-17-Producing cells in the affected areas of the skin. Case Rep Dermatol (2012) 4(3):211–4. doi: 10.1159/000343882 PMC3493012 23139659

[17] Lee YY Bee PC Lee CK Naiker M Ismail R. Bullous pemphigoid in an elderly patient with myelodysplastic syndrome and refractory anemia coupled with excess of blast. Ann Dermatol (2011) 23(Suppl 3):S390–2. doi: 10.5021/ad.2011.23.S3.S390 PMC3276805 22346286

[18] Schulze F Neumann K Recke A Zillikens D Linder R Schmidt E. Malignancies in pemphigus and pemphigoid diseases. J Invest Dermatol (2015) 135(5):1445–7. doi: 10.1038/jid.2014.547 25560279

[19] Atzmony L Mimouni I Reiter O Leshem YA Taha O Gdalevich M. Association of bullous pemphigoid with malignancy: A systematic review and meta-analysis. J Am Acad Dermatol (2017) 77(4):691–9. doi: 10.1016/j.jaad.2017.05.006 28645646

[20] Cai SC Allen JC Lim YL Chua SH Tan SH Tang MB. Mortality of bullous pemphigoid in Singapore: risk factors and causes of death in 359 patients seen at the national skin centre. Br J Dermatol (2014) 170(6):1319–26. doi: 10.1111/bjd.12806 24372558

[21] Ma H Chang H. Life-threatening bleeding in a patient with pemphigoid-induced acquired hemophilia a and successfully treated with rituximab and rFVIIa: A case report. Medicine (2021) 100(3):e24025. doi: 10.1097/MD.0000000000024025 33545998 PMC7837823

[22] Braganca M Valente C Ferreira AI Freitas-Silva M. Acquired hemophilia a associated with bullous pemphigoid: A rare combination. Transfus Apher Sci (2021) 61(2):103337. doi: 10.1016/j.transci.2021.103337 34903450

[23] Binet Q Lambert C Sacre L Eeckhoudt S Hermans C. Successful management of acquired hemophilia a associated with bullous pemphigoid: A case report and review of the literature. Case Rep Hematol (2017) 2017:2057019. doi: 10.1155/2017/2057019 28458935 PMC5387803

[24] Aljasser MI Sladden C Crawford RI Au S. Bullous pemphigoid associated with acquired hemophilia a: a rare association of autoimmune disease. J Cutan Med Surg (2014) 18(2):123–6. doi: 10.2310/7750.2013.13060 24636438

[25] Makita S Aoki T Watarai A Aida A Katayama T Danbara M. Acquired hemophilia associated with autoimmune bullous diseases: a report of two cases and a review of the literature. Intern Med (2013) 52(7):807–10. doi: 10.2169/internalmedicine.52.9317 23545680

[26] Ammannagari N Laveaux K Grethlein S. Acquired hemophilia in the setting of bullous pemphigoid: A case report. J Hematol (2013) 2(2):74–5. doi: 10.4021/jh74w

[27] Qiu X Zhang G Xiao R Zhang J Zhou Y Li G. Acquired hemophilia associated with bullous pemphigoid: a case report. Int J Clin Exp Pathol (2012) 5(1):102–4. PMC3267493 22295154

[28] Zhang X Guo J Guo X Pan J. Successful treatment of acquired haemophilia in a patient with bullous pemphigoid with single-dosing regimen of rituximab. Haemophilia. (2012) 18(5):e393–5. doi: 10.1111/j.1365-2516.2012.02917.x 22823057

[29] Nguyen C Gordon JS Chang AL. A little known but potentially life-threatening association of bullous pemphigoid and acquired hemophilia: Case report and review of the literature. J Clin Exp Dermatol Res (2012) S6:1–3. doi: 10.4172/2155-9554.S6-003

[30] Kluger N Navarro R Pallure V Guillot B. [Bullous pemphigoid and acquired haemophilia]. Ann Dermatol Venereol (2011) 138(5):422–3. doi: 10.1016/j.annder.2011.01.040 21570569

[31] Chen C Chen Y Ho J Wu C. Bullous pemphigoid associated with acquired hemophilia. Dermatologica Sinica (2010) 28(4):173–6. doi: 10.1016/S1027-8117(10)60038-9

[32] Maczek C Thoma-Uszynski S Schuler G Hertl M. [Simultaneous onset of pemphigoid and factor VIII antibody hemophilia]. Hautarzt. (2002) 53(6):412–5. doi: 10.1007/s001050100278 12132299

[33] Aoki Y Miyake N Yamasowa M Inoue F Takamatsu T Mizumoto T. A case of autoimmune hemolytic anemia and bullous pemphigoid-like skin lesion combined with idiopathic thrombocytopenic purpura. Rinsho Ketsueki (1990) 31(3):346–51. 2195183

[34] Wang FJ Liu HH Kong XX Wu MJ. Report on a bullous pemphigoid case manifested as eosinophilia dermatitis. Chin J Lepr Skin Dis (2017) 33:37–8.

[35] Belgnaoui F Idrissi M Benyoussef K Loudiye T Bella A Senouci K. Idiopathic hypereosinophilic syndrome and bullous pemphigoid. Ann Dermatol Venereol (2002) 129(11):1291–4. 12514518

[36] Bauduer F Barteau A Truchet S Massot-Bordenave J Ducout L. Bullous pemphigoid associated with the transformation of a preexisting myelodysplastic syndrome. Leuk Lymphoma. (1999) 32(3-4):399–400. doi: 10.3109/10428199909167405 10037042

[37] Modiano P Reichert S Barbaud A Bernard P Weber M Schmutz JL. Bullous pemphigoid in association with cutaneous lesions specific to a myelodysplastic syndrome. Br J Dermatol (1997) 136(3):402–5. 9115926

[38] Ivars M Hashimoto T Ishii N Bernad I Lecumberri R Espana A. Atypical bullous pemphigoid with extensive cutaneous and mucosal erosions associated with chronic lymphocytic leukemia. J Dermatol (2015) 42(11):1128–9. doi: 10.1111/1346-8138.13067 26299375

[39] Kassim JM Igali L Levell NJ. A 14-year paraneoplastic rash: urticarial vasculitis and dermal binding bullous pemphigoid secondary to chronic lymphocytic leukaemia. Clin Exp Dermatol (2015) 40(4):391–4. doi: 10.1111/ced.12553 25524180

[40] Kakurai M Demitsu T Iida E Umemoto N Yamada T Yoneda K. Coexistence of paraneoplastic pemphigus and bullous pemphigoid. J Eur Acad Dermatol Venereol (2009) 23(8):962–4. doi: 10.1111/j.1468-3083.2008.03071.x 19207671

[41] Saouli Z Papadopoulos A Kaiafa G Girtovitis F Kontoninas Z. A new approach on bullous pemphigoid therapy. Ann Oncol (2008) 19(4):825–6. doi: 10.1093/annonc/mdn046 18325915

[42] Ameen M Pembroke AC Black MM Russell-Jones R. Eosinophilic spongiosis in association with bullous pemphigoid and chronic lymphocytic leukaemia. Br J Dermatol (2000) 143(2):421–4. doi: 10.1046/j.1365-2133.2000.03674.x 10951157

[43] Misery L Cambazard F Rimokh R Ghohestani R Magaud JP Gaudillere A. Bullous pemphigoid associated with chronic b-cell lymphatic leukaemia: the anti-230-kDa autoantibody is not synthesized by leukaemic cells. Br J Dermatol (1999) 141(1):155–7. doi: 10.1046/j.1365-2133.1999.02940.x 10417535

[44] Su WP Oursler JR Muller SA. Paraneoplastic pemphigus: a case with high titer of circulating anti-basement membrane zone autoantibodies. J Am Acad Dermatol (1994) 30(5 Pt 2):841–4. doi: 10.1016/s0190-9622(94)70093-1 8169257

[45] Goodnough LT Muir WA. Bullous pemphigoid as a manifestation of chronic lymphocytic leukemia. Arch Intern Med (1980) 140(11):1526–7. doi: 10.1001/archinte.1980.00330220077028 7002083

[46] Cuni LJ Grunwald H Rosner F. Bullous pemphigoid in chronic lymphocytic leukemia with the demonstration of antibasement membrane antibodies. Am J Med (1974) 57(6):987–92. doi: 10.1016/0002-9343(74)90179-x 4611210

[47] Godaert L Bartholet S Colas S Kanagaratnam L Fanon JL Drame M. Acquired hemophilia a in aged people: A systematic review of case reports and case series. Semin Hematol (2018) 55(4):197–201. doi: 10.1053/j.seminhematol.2018.02.004 30502847

[48] Windyga J Baran B Odnoczko E Buczma A Drews K Laudanski P. Treatment guidelines for acquired hemophilia a. Ginekol Pol (2019) 90(6):353–64. doi: 10.5603/GP.2019.0063 31276188

[49] Patel RS Harman KE Nichols C Burd RM Pavord S. Acquired haemophilia heralded by bleeding into the oral mucosa in a patient with bullous pemphigoid, rheumatoid arthritis, and vitiligo. Postgrad Med J (2006) 82(963):e3. doi: 10.1136/pgmj.2005.036483 16397069 PMC2563721

[50] Zuo WL Zhang GS Qing ZJ Xu YX Qin LX Xu M. Identification of IgG subclass and FVIII binding epitope of an acquired FVIII inhibitor in a bullous pemphigoid patient. Zhonghua Xue Ye Xue Za Zhi (2006) 27(9):593–7. doi: 10.3760/cma.j.issn.0253-2727.2006.09.006 17278424

[51] Marzano AV Tedeschi A Fanoni D Bonanni E Venegoni L Berti E. Activation of blood coagulation in bullous pemphigoid: role of eosinophils, and local and systemic implications. Br J Dermatol (2009) 160(2):266–72. doi: 10.1111/j.1365-2133.2008.08880.x 18945300

[52] Lin L Hwang BJ Culton DA Li N Burette S Koller BH. Eosinophils mediate tissue injury in the autoimmune skin disease bullous pemphigoid. J Invest Dermatol (2018) 138(5):1032–43. doi: 10.1016/j.jid.2017.11.031 PMC7531612 29246800

[53] Eisenbarth GS Gottlieb PA. Autoimmune polyendocrine syndromes. N Engl J Med (2004) 350(20):2068–79. doi: 10.1056/NEJMra030158 15141045

[54] Mangin MA Lienhart A Gouraud A Roux S Hodique F Jouen F. Onset of acquired haemophilia a after omalizumab treatment in severe bullous pemphigoid - a report on two cases successfully treated with mycophenolate mofetil. Ann Dermatol Venereol (2021) 148(1):57–9. doi: 10.1016/j.annder.2020.09.577 33461795

[55] Sourdeau E Clauser S Prud’Homme R Bardet V Calmette L. Acquired hemophilia a associated with bullous pemphigoid and multiple myeloma: a case report. Ann Biol Clin (Paris) (2019) 77(2):179–83. doi: 10.1684/abc.2018.1405 30882350

[56] Sugiyama S Tanaka R Hayashi H Izumi K Nishie W Aoyama Y. Acquired haemophilia a in DPP4 inhibitor-induced bullous pemphigoid as immune reconstitution syndrome. Acta Derm Venereol (2020) 100(13):adv00178. doi: 10.2340/00015555-3539 32494825 PMC9175053

[57] Kruse-Jarres R Kempton CL Baudo F Collins PW Knoebl P Leissinger CA. Acquired hemophilia a: Updated review of evidence and treatment guidance. Am J Hematol (2017) 92(7):695–705. doi: 10.1002/ajh.24777 28470674

[58] Collins PW Hirsch S Baglin TP Dolan G Hanley J Makris M. Acquired hemophilia a in the united kingdom: a 2-year national surveillance study by the united kingdom haemophilia centre doctors’ organisation. Blood. (2007) 109(5):1870–7. doi: 10.1182/blood-2006-06-029850 17047148

[59] Muhammed N Korgaonkar S Pradhan V Khopkar US. A cross-sectional study to correlate disease severity in bullous pemphigoid patients with serum levels of autoantibodies against BP180 and BP230. Indian Dermatol Online J (2021) 12(5):696–700. doi: 10.4103/idoj.IDOJ_813_20 34667755 PMC8456256

[60] Lambert MP Gernsheimer TB. Clinical updates in adult immune thrombocytopenia. Blood. (2017) 129(21):2829–35. doi: 10.1182/blood-2017-03-754119 PMC5813736 28416506

[61] Bakchoul T Marini I. Drug-associated thrombocytopenia. Hematol Am Soc Hematol Educ Program (2018) 2018(1):576–83. doi: 10.1182/asheducation-2018.1.576 PMC6246020 30504360

[62] Noh HR Magpantay GG. Hypereosinophilic syndrome. Allergy Asthma Proc (2017) 38(1):78–81. doi: 10.2500/aap.2017.38.3995 28052805

[63] Kahn JE Girszyn N Bletry O. Diagnosis of non-parasitic hypereosinophilia. Presse Med (2006) 35(1 Pt 2):144–52. doi: 10.1016/s0755-4982(06)74537-7 16462679

[64] Muller AM Martens UM Hofmann SC Bruckner-Tuderman L Mertelsmann R Lubbert M. Imatinib mesylate as a novel treatment option for hypereosinophilic syndrome: two case reports and a comprehensive review of the literature. Ann Hematol (2006) 85(1):1–16. doi: 10.1007/s00277-005-1084-7 16136348

[65] Kridin K. Peripheral eosinophilia in bullous pemphigoid: prevalence and influence on the clinical manifestation. Br J Dermatol (2018) 179(5):1141–7. doi: 10.1111/bjd.16679 29663327

[66] Verraes S Hornebeck W Polette M Borradori L Bernard P. Respective contribution of neutrophil elastase and matrix metalloproteinase 9 in the degradation of BP180 (type XVII collagen) in human bullous pemphigoid. J Invest Dermatol (2001) 117(5):1091–6. doi: 10.1046/j.0022-202x.2001.01521.x 11710917

[67] Amber KT Valdebran M Kridin K Grando SA. The role of eosinophils in bullous pemphigoid: A developing model of eosinophil pathogenicity in mucocutaneous disease. Front Med (2018) 5:201. doi: 10.3389/fmed.2018.00201 PMC6048777 30042946

[68] Wakugawa M Nakamura K Hino H Toyama K Hattori N Okochi H. Elevated levels of eotaxin and interleukin-5 in blister fluid of bullous pemphigoid: correlation with tissue eosinophilia. Br J Dermatol (2000) 143(1):112–6. doi: 10.1046/j.1365-2133.2000.03599.x 10886144

[69] Saniklidou AH Tighe PJ Fairclough LC Todd I. IgE autoantibodies and their association with the disease activity and phenotype in bullous pemphigoid: a systematic review. Arch Dermatol Res (2018) 310(1):11–28. doi: 10.1007/s00403-017-1789-1 29071428 PMC5754504

[70] Young NS. Aplastic anemia. N Engl J Med (2018) 379(17):1643–56. doi: 10.1056/NEJMra1413485 PMC6467577 30354958

[71] Bettelli E Oukka M Kuchroo VK. T(H)-17 cells in the circle of immunity and autoimmunity. Nat Immunol (2007) 8(4):345–50. doi: 10.1038/ni0407-345 17375096

[72] de Latour RP Visconte V Takaku T Wu C Erie AJ Sarcon AK. Th17 immune responses contribute to the pathophysiology of aplastic anemia. Blood. (2010) 116(20):4175–84. doi: 10.1182/blood-2010-01-266098 PMC2993623 20733158

[73] Arakawa M Dainichi T Ishii N Hamada T Karashima T Nakama T. Lesional Th17 cells and regulatory T cells in bullous pemphigoid. Exp Dermatol (2011) 20(12):1022–4. doi: 10.1111/j.1600-0625.2011.01378.x 22017210

[74] Hasserjian RP. Myelodysplastic syndrome updated. Pathobiology. (2019) 86(1):7–13. doi: 10.1159/000489702 30041243

[75] Green KJ Virata ML Elgart GW Stanley JR Parry DA. Comparative structural analysis of desmoplakin, bullous pemphigoid antigen and plectin: members of a new gene family involved in organization of intermediate filaments. Int J Biol Macromol (1992) 14(3):145–53. doi: 10.1016/s0141-8130(05)80004-2 1390446

[76] Venning VA Wojnarowska F. The association of bullous pemphigoid and malignant disease: a case control study. Br J Dermatol (1990) 123(4):439–45. doi: 10.1111/j.1365-2133.1990.tb01447.x 2095174

[77] Iranzo P Lopez I Robles MT Mascaro JM Jr. Campo E Herrero C. Bullous pemphigoid associated with mantle cell lymphoma. Arch Dermatol (2004) 140(12):1496–9. doi: 10.1001/archderm.140.12.1496 15611428

[78] Jung M Rice L. Unusual autoimmune nonhematologic complications in chronic lymphocytic leukemia. Clin Lymphoma Myeloma Leuk (2011) 11 Suppl 1:S10–3. doi: 10.1016/j.clml.2011.02.005 22035737

[79] Maglie R Genovese G Solimani F Guglielmo A Pileri A Portelli F. Immune-mediated dermatoses in patients with haematological malignancies: A comprehensive review. Am J Clin Dermatol (2020) 21(6):833–54. doi: 10.1007/s40257-020-00553-9 PMC7679319 32813229

[80] Ong E Goldacre R Hoang U Sinclair R Goldacre M. Associations between bullous pemphigoid and primary malignant cancers: an English national record linkage study, 1999-2011. Arch Dermatol Res (2014) 306(1):75–80. doi: 10.1007/s00403-013-1399-5 23912480

[81] Maglie R Ugolini F De Logu F Nassini R Simi S Nardiello P. Overexpression of helper T cell type 2-related molecules in the skin of patients with eosinophilic dermatosis of hematologic malignancy. J Am Acad Dermatol (2022) 87(4):761–70. doi: 10.1016/j.jaad.2021.07.007 34265409

[82] Qiao J Sun CE Zhu W Zhu D Fang H. Flame figures associated with eosinophilic dermatosis of hematologic malignancy: is it possible to distinguish the condition from eosinophilic cellulitis in patients with hematoproliferative disease? Int J Clin Exp Pathol (2013) 6(8):1683–7. PMC3726987 23923089

[83] Maglie R Antiga E Vannucchi M Del Bianco E Bianchi B Massi D. Bullous eruption in a patient with b-cell chronic lymphocytic leukemia: a diagnostic challenge. Int J Dermatol (2017) 56(12):1445–7. doi: 10.1111/ijd.13807 29076242

[84] Ouedraogo E Gottlieb J de Masson A Lepelletier C Jachiet M Salle de Chou C. Risk factors for death and survival in paraneoplastic pemphigus associated with hematologic malignancies in adults. J Am Acad Dermatol (2019) 80(6):1544–9. doi: 10.1016/j.jaad.2018.03.043 30981429

[85] Huang Y Li J Zhu X. Detection of anti-envoplakin and anti-periplakin autoantibodies by ELISA in patients with paraneoplastic pemphigus. Arch Dermatol Res (2009) 301(10):703–9. doi: 10.1007/s00403-008-0901-y 18820940

[86] Avalos AM Meyer-Wentrup F Ploegh HL. B-cell receptor signaling in lymphoid malignancies and autoimmunity. Adv Immunol (2014) 123:1–49. doi: 10.1016/B978-0-12-800266-7.00004-2 24840946

[87] Kato K Koike K Kobayashi C Iijima S Hashimoto T Tsuchida M. Bullous pemphigoid after allogeneic hematopoietic stem cell transplantation. Pediatr Int (2015) 57(3):480–3. doi: 10.1111/ped.12561 26113316

[88] Maverakis E Goodarzi H Wehrli LN Ono Y Garcia MS. The etiology of paraneoplastic autoimmunity. Clin Rev Allergy Immunol (2012) 42(2):135–44. doi: 10.1007/s12016-010-8248-5 21246308

[89] De Matteis S Molinari C Abbati G Rossi T Napolitano R Ghetti M. Immunosuppressive treg cells acquire the phenotype of effector-T cells in chronic lymphocytic leukemia patients. J Transl Med (2018) 16(1):172. doi: 10.1186/s12967-018-1545-0 29925389 PMC6011245

[90] Balestri R Magnano M La Placa M Patrizi A Angileri L Tengattini V. Malignancies in bullous pemphigoid: A controversial association. J Dermatol (2016) 43(2):125–33. doi: 10.1111/1346-8138.13079 26435381

[91] Robak E Robak T. Skin lesions in chronic lymphocytic leukemia. Leuk Lymphoma. (2007) 48(5):855–65. doi: 10.1080/10428190601137336 17487727

